# Effects of Varying Epoch Lengths, Wear Time Algorithms, and Activity Cut-Points on Estimates of Child Sedentary Behavior and Physical Activity from Accelerometer Data

**DOI:** 10.1371/journal.pone.0150534

**Published:** 2016-03-03

**Authors:** Jorge A. Banda, K. Farish Haydel, Tania Davila, Manisha Desai, Susan Bryson, William L. Haskell, Donna Matheson, Thomas N. Robinson

**Affiliations:** 1 Stanford Solutions Science Lab, Department of Pediatrics and Stanford Prevention Research Center, Stanford University School of Medicine, Stanford, California, United States of America; 2 Stanford Prevention Research Center, Department of Medicine, Stanford University School of Medicine, Stanford, California, United States of America; 3 Division of General Pediatrics, Department of Pediatrics, Stanford University School of Medicine, Palo Alto, California, United States of America; 4 Quantitative Sciences Unit, Department of Medicine, Stanford University School of Medicine, Palo Alto, California, United States of America; University of Catania, ITALY

## Abstract

**Objective:**

To examine the effects of accelerometer epoch lengths, wear time (WT) algorithms, and activity cut-points on estimates of WT, sedentary behavior (SB), and physical activity (PA).

**Methods:**

268 7–11 year-olds with BMI ≥ 85^th^ percentile for age and sex wore accelerometers on their right hips for 4–7 days. Data were processed and analyzed at epoch lengths of 1-, 5-, 10-, 15-, 30-, and 60-seconds. For each epoch length, WT minutes/day was determined using three common WT algorithms, and minutes/day and percent time spent in SB, light (LPA), moderate (MPA), and vigorous (VPA) PA were determined using five common activity cut-points. ANOVA tested differences in WT, SB, LPA, MPA, VPA, and MVPA when using the different epoch lengths, WT algorithms, and activity cut-points.

**Results:**

WT minutes/day varied significantly by epoch length when using the NHANES WT algorithm (p < .0001), but did not vary significantly by epoch length when using the ≥ 20 minute consecutive zero or Choi WT algorithms. Minutes/day and percent time spent in SB, LPA, MPA, VPA, and MVPA varied significantly by epoch length for all sets of activity cut-points tested with all three WT algorithms (all p < .0001). Across all epoch lengths, minutes/day and percent time spent in SB, LPA, MPA, VPA, and MVPA also varied significantly across all sets of activity cut-points with all three WT algorithms (all p < .0001).

**Conclusions:**

The common practice of converting WT algorithms and activity cut-point definitions to match different epoch lengths may introduce significant errors. Estimates of SB and PA from studies that process and analyze data using different epoch lengths, WT algorithms, and/or activity cut-points are not comparable, potentially leading to very different results, interpretations, and conclusions, misleading research and public policy.

## Introduction

Accelerometers are commonly used to objectively measure sedentary behavior (SB) and physical activity (PA), including assessing activity levels for population level surveillance, examining associations between activity levels and measures of health and disease, and examining the efficacy of PA promotion and obesity prevention and treatment efforts. Researchers make many data processing and analysis decisions when creating accelerometer-derived estimates of SB and PA [1]. One key decision is the choice of epoch length, which refers to the interval of time over which the units of accelerometer measures (also known as “counts”) are summed. Another important decision is the selection of an algorithm to differentiate accelerometer wear time (WT) from non-wear time. A third decision is the selection of activity cut-point definitions to determine the amount of time spent in SB and PA intensity levels. Of particular interest is whether it is acceptable to use an epoch length for data analysis that differs from the epoch length originally used to validate algorithms and activity cut-point definitions for WT, SB, and PA intensity levels, and whether results vary when using different validated WT algorithms and activity cut-points.

In practice, researchers (including those in our research lab) have often converted WT algorithms and activity cut-point definitions to match the epoch length used to collect and/or analyze accelerometer data [2]. For example, although the U.S. National Health and Nutrition Examination Survey (NHANES) WT algorithm was developed for 60-second epoch data [3], the WT algorithm is commonly applied to data with different epoch lengths. Previously validated activity cut-points are often converted from their validated epoch length to match other epoch lengths. For example, the SB activity cut-point of 100 counts/60-second epoch is sometimes converted to 50 counts/30-second epoch or 25 counts/15-second epoch. Although this practice appears reasonable on the surface, the validity of converting WT algorithms and activity cut-point definitions to different epoch lengths is unknown. Current generation accelerometers that can collect acceleration data at very high frequencies (allowing activity counts to be reintegrated into any desired epoch length after data collection) allow the validity of this common practice to be tested.

Research has begun to examine the effects of epoch length on accelerometer-derived measures. One study [4] examined the effect of epoch length on WT, finding no significant difference in WT hours/day by epoch length when using a ≥ 10 minute consecutive zero vertical-axis count WT algorithm. It reasons, however, that epoch length may have an effect on WT for algorithms that include an allowance period for artificial movements, such as the NHANES [3] and Choi and colleagues [5] WT algorithms, due to the way non-wear time is accumulated with different epoch lengths. Significant differences have been observed in time spent in SB and PA intensity levels by epoch length among children 7 years and older [4, 6–9]. However, studies in this literature have used a limited number of activity cut-points to determine time spent in SB and PA intensity levels, and it remains unclear whether the differences observed by epoch length in these studies generalize to other standard activity cut-points. The purpose of the current study was to examine the effects of varying accelerometer epoch lengths, WT algorithms, and activity cut-point definitions on estimates of WT, SB, and PA intensity levels. This study used accelerometer data from a large, community-based sample of children with overweight and obesity.

## Methods

### Participants

This study uses baseline data from the Stanford GOALS trial [10]. Participants were recruited from low-income, primarily Latino neighborhoods in Northern California and were 7–11 years of age with a body mass index (BMI) ≥ 85^th^ percentile for age and sex. A parent or guardian provided written consent and HIPAA authorization, and children gave assent to participate in the study. The study was approved by the Stanford University Administrative Panel on Human Subjects in Medical Research and received oversight from an independent Data and Safety Monitory Board formed by the National Heart, Lung, and Blood Institute.

### Participant Measures

Participant demographic characteristics, height, and weight were collected according to standardized protocols [10]. The ActiGraph GT3X+ monitor (ActiGraph, Pensacola, FL) was used to measure movement. Participants were instructed to wear the monitor on a belt on their right hip for seven complete days, including while sleeping, except during water activities (e.g., bathing, swimming, showering). Participants were asked to re-wear the monitor if they did not meet minimum WT requirements (3 weekdays and 1 weekend day with ≥ 360 WT minutes/day).

### Accelerometer Data Processing

The monitor measures accelerations in three individual axes (vertical, horizontal, perpendicular), has a dynamic range of ± 6 units of gravity, and was set to record at a frequency of 40Hz (i.e., collect acceleration data 40 times per second in each axis). ActiLife version 6.10.2 was used to download these data from the monitor, and to convert acceleration data into vertical-axis and vector magnitude activity counts at epoch lengths of 1-, 5-, 10-, 15-, 30-, and 60-seconds. The ActiLife wear time validation program was used to determine daily WT minutes for each of these epoch lengths using three different WT algorithms commonly used in children (Table 1): the ≥ 20 minute consecutive zero vertical-axis count WT algorithm [1], the NHANES WT algorithm [3, 11], and the Choi and colleagues WT algorithm [5].

**Table 1:** Accelerometer WT algorithms.

≥ 20 Minute Consecutive Zero Vertical-Axis Count WT Algorithm [1]	NHANES WT Algorithm [3]	Choi WT Algorithm [5]
*Non-wear* was defined by at least 20 consecutive minutes of zero counts. **Derived Epoch Length**: Not Available.	*Non-wear* was defined by an interval of at least 60 consecutive minutes of zero activity intensity counts, with allowance for 1–2 minutes of counts between 0 and 100. **Note**: This study used a 2-minute allowance [11] of counts between 0 and 100. **Derived Epoch Length**: 60-second.	1-minute time intervals with consecutive zero counts for a time window of at least 90-minutes (window 1), allowing intervals with non-zero counts lasting up to 2-minutes (allowance interval) if no counts are detected during both the 30-minutes upstream and downstream from that interval (window 2); any non-zero counts except the allowed short interval are considered as wear time. **Derived Epoch Length**: 60-second.

The ActiLife data scoring program was used to determine daily minutes spent in SB, light PA (LPA), moderate PA (MPA), vigorous PA (VPA), and moderate and vigorous PA (MVPA) between 5:00AM and 11:59PM for each epoch length dataset (excluding non-wear time), using the Evenson [12], Treuth [13], Puyau [14], Mattocks [15], and Romanzini [16] activity cut-points (Table 2). Activity cut-points were converted to match each epoch length tested (e.g., 100 counts/60-second epoch was converted to 25 counts/15-second epoch). Percent time spent in SB, LPA, MPA, VPA, and MVPA was calculated using author-written SAS programs. Days with ≥ 360 WT minutes when examined with 60-second epoch length data were included in the analysis. These steps were repeated for each WT validation algorithm.

**Table 2:** Accelerometer activity cut-points from original validation studies.

Activity Cut-Points	Axis	Years of Age	Sex	Epoch Length used in Validation Study	SB	LPA	MPA	VPA
**Evenson** [12]	Vertical Axis	5–9	Boys and Girls	15-second	0–25	26–573	574–1002	≥ 1003
**Treuth** [13]	Vertical Axis	13–15	Girls	30-second	0–50	51–1499	1500–2600	≥ 2601
**Puyau** [14]	Vertical Axis	6–16	Boys and Girls	60-second	0–799	800–3199	3200–8199	≥ 8200
**Mattocks** [15] *	Vertical Axis	12	Boys and Girls	60-second	0–3580		3581–6129	≥ 6130
**Romanzini** [16]	Vector Magnitude	10–15	Boys and Girls	15-second	0–180	181–756	757–1111	≥ 1112

SB = Sedentary behavior, LPA = Light physical activity, MPA = Moderate physical activity, VPA = Vigorous physical activity

* The Mattocks activity cut-point does not provide separate activity cut-points for SB and LPA.

### Statistical Analysis

First, F tests from Analysis of Variance (ANOVA) models were used to assess whether WT, SB, LPA, MPA, VPA, and MVPA estimates varied by epoch length within each WT algorithm and activity cut-point, with statistical significance defined using the conservative Bonferroni correction method to account for multiple tests (α = 0.05/11 = 0.0045). Post hoc analyses were then used to identify pairwise differences in WT estimates between the epoch length used to validate the WT algorithm (e.g., 60-second epoch length for the NHANES WT algorithm) and other epoch lengths (e.g., 1-, 5-, 10-, 15-, and 30-second epoch lengths). Post hoc analyses were also used to identify pairwise differences in SB, LPA, MPA, VPA, and MVPA estimates between the epoch length used to validate the activity cut-point (e.g., 15-second epoch length for the Evenson activity cut-points) and other epoch lengths (e.g., 1-, 5-, 10-, 30-, and 60-second epoch lengths) within each WT algorithm.

Second, F tests from Analysis of Variance (ANOVA) models were used to assess whether SB, LPA, MPA, VPA, and MVPA estimates varied by activity cut-point, using the epoch length used to validate each activity cut-point (see Table 2) within each WT algorithm, with statistical significance defined using the Bonferroni correction method to account for multiple tests (α = 0.05/10 = 0.005). Post hoc analyses were then used to identify pairwise differences in SB, LPA, MPA, VPA, and MVPA estimates between activity cut-points. Mattocks activity cut-point determined SB and LPA were excluded from the activity cut-point analysis, as the Mattocks activity cut-point does not provide separate cut-points for SB and LPA. Statistical analysis was performed using SAS version 9.4 (SAS Institute Inc., Cary, North Carolina).

## Results

### Participant Characteristics

Participants (N = 268 children in 241 families) had a mean age of 9.53 ± 1.46 years, mean BMI percentile of 96.45 ± 3.27, were 54.9% girls, and were primarily Hispanic or Latino (98%). Participants had a mean ± SD of 7.2 ± 1.2 days with ≥ 360 WT minutes when examined with 60-second epoch length data and the ≥ 20 minute consecutive zero vertical-axis count WT algorithm, 7.3 ± 1.2 days with ≥ 360 WT minutes with the NHANES WT algorithm, and 7.4 ± 1.2 days with ≥ 360 WT minutes with the Choi WT algorithm.

### Estimates of WT by Epoch Length

**Fig 1:**
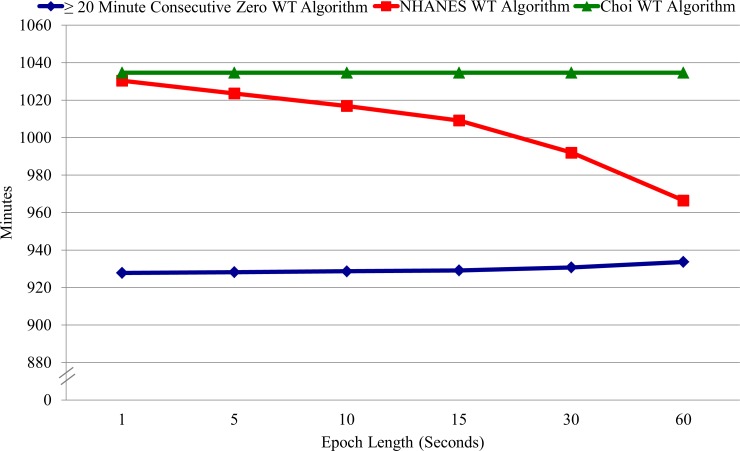
Accelerometer WT minutes/day by WT algorithm and epoch length.

WT minutes/day are shown in Fig 1, and are available as online supporting information (S1–S3 Tables). When using the ≥ 20 minute consecutive zero vertical-axis count WT algorithm, WT minutes/day did not significantly vary by epoch length (p = .684), ranging from 927.77 minutes/day (1-second epoch) to 933.66 minutes/day (60-second epoch). Similarly, when using the Choi WT algorithm, WT minutes/day did not vary by epoch length (p > 0.99), as WT minutes/day were identical for all epoch lengths (1034.65 minutes/day). When using the NHANES WT algorithm, however, WT minutes/day varied significantly by epoch length (p < .0001), ranging from 1030.33 minutes/day (1-second epoch) to 966.44 minutes/day (60-second epoch). Pair-wise comparisons for WT minutes/day between the epoch length used to validate the NHANES WT algorithm (60-second epoch) and other epoch lengths (1-, 5-, 10-, 15-, and 30-second epochs) suggested significant differences with all other epoch lengths (all p values < .0001).

### Estimates of SB and PA Levels by Epoch Length, WT Algorithm, and Activity Cut-Points

**Fig 2:**
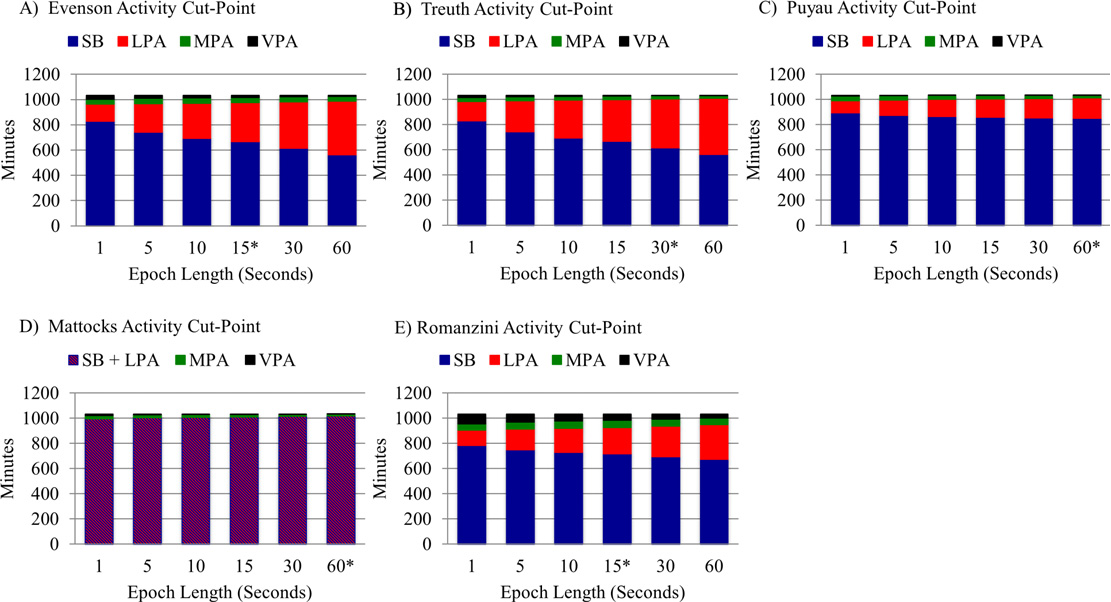
Minutes/day spent in SB and PA by epoch length using the Choi WT algorithm. *Derived epoch length used in the original validation study.

Minutes/day and percent time spent in SB, LPA, MPA, VPA, and MVPA varied significantly by epoch length when using the Evenson, Treuth, Puyau, Mattocks, and Romanzini activity cut-points with all three WT algorithms (all p values < .0001) (S1–S3 Tables). A vast majority (97%) of pairwise comparisons for minutes/day and percent time spent in SB, LPA, MPA, VPA, and MVPA from epoch lengths used to validate activity cut-points with other epoch lengths suggested significant differences (p < .05) for the Evenson, Treuth, Puyau, Mattocks, and Romanzini activity cut-points when using all three WT algorithms (S1–S3 Tables). These results are illustrated in Fig 2 using the Choi WT algorithm, in which WT does not vary by epoch length so the effects of epoch length on SB and PA levels are isolated.

### Differences in Estimates of SB and PA Levels by Activity Cut-Points

**Fig 3:**
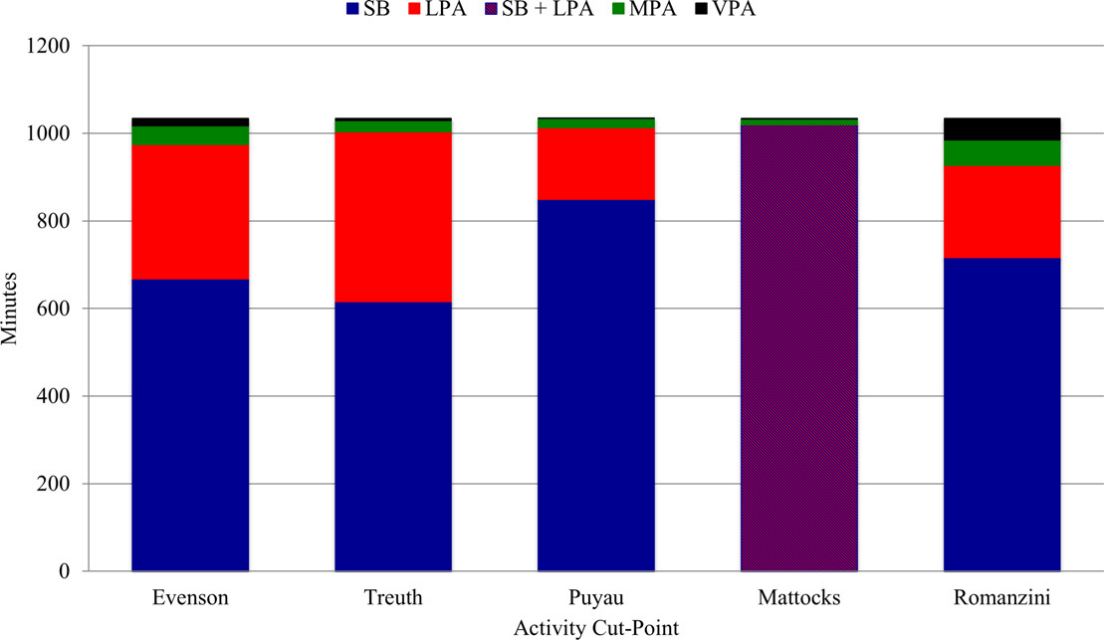
Minutes/day spent in SB and PA by activity cut-point using the Choi WT algorithm. The epoch length originally used to validate the activity cut-points were used to determine estimates of SB and PA.

Minutes/day and percent time spent in SB, LPA, MPA, VPA, and MVPA varied significantly by activity cut-point when using all three WT algorithms (all p values < .0001) (S4–S6 Tables). All pairwise comparisons for minutes/day and percent time spent in SB, LPA, MPA, VPA, and MVPA between activity cut-points suggested significant differences (all p values < .05; 95% of pairwise comparisons had a p value < .0001) when using all three WT algorithms (S4–S6 Tables). These results are illustrated in Fig 3 using the Choi WT algorithm, in which WT does not vary by epoch length so the effects of activity cut-point alone are isolated.

## Discussion

In this study we discovered problems when comparing data analyzed at different epoch lengths, particularly the common practice of converting WT algorithms and activity cut-point definitions to different epoch lengths. As a result, using epoch lengths that differ from those originally used to validate the WT algorithms and activity cut-points introduces significant errors into resulting estimates of WT, SB, and PA intensity levels. In addition, we discovered that different activity cut-points, although all validated, can produce very different estimates of SB and PA levels, even when using the epoch lengths used in their validation studies.

### Estimates of WT by Epoch Length

There was no significant effect of epoch length on WT with the ≥ 20 minute consecutive zero vertical-axis count WT algorithm. This was expected due to the algorithm not having an allowance period and is consistent with previous research using a ≥ 10 minute consecutive zero vertical-axis count WT algorithm [4].

The significant effect of epoch length on WT with the NHANES WT algorithm could have been expected due to the way non-wear time is accumulated with allowance periods through different epoch lengths. For example, 7 counts occurring during a 10-second epoch during NHANES-determined non-wear time would contribute only 10-seconds towards a 2-minute allowance period. However, if data were reintegrated into a 60-second epoch, these same counts would now contribute 60-seconds towards a 2-minute allowance period. We were unable to identify prior studies examining the effect of epoch length on WT using the NHANES WT algorithm.

The Choi WT algorithm also uses an allowance period, but there was no observed effect of epoch length on WT. These results first seemed counterintuitive. However, these results can be explained by functions in the “PhysicalActivity” R package developed by Choi and colleagues [17]. Before the Choi WT algorithm is applied, the “wearingMarking” function collapses all non 60-second epoch data into 60-second epoch data. For example, the 1-, 5-, 10-, 15-, and 30-second epoch data in this study would be collapsed into 60-second epoch data, and the Choi WT algorithm would then be applied to the 60-second epoch datasets. As the Choi WT algorithm is only applied to 60-second epoch datasets, the resulting WT minutes/day estimate is the same regardless of the original (pre-collapsed) epoch length used. We applied the Choi WT algorithm to a sub-sample of participants using the “PhysicalActivity” R package and the ActiLife software. The results show both programs function in the same way to determine WT minutes/day. As the output data sets are in their original (pre-collapsed) epoch length, the effects of epoch length on estimates of SB and PA intensity levels can still be examined.

### Estimates of SB and PA by Epoch Length

Similar patterns of results were observed for minutes/day and percent time spent in SB and PA intensity levels for all three WT algorithms used in this study. As epoch lengths increased, estimates of SB, MPA, and VPA decreased, whereas estimates of LPA increased. An interesting relationship was observed between SB and LPA by epoch length, as a large amount of SB was reclassified as LPA when larger epoch lengths were used, regardless of the activity cut-point definitions used. This relationship is clearly visible in Fig 2.

The differences in estimates observed in the SB and MVPA results may be particularly troublesome in practice because they play a prominent role in clinical and public health recommendations for PA among youth. For example, in this sample of children with overweight and obesity the mean percent of time spent in SB when calculated with the Evenson cut-points and Choi WT algorithm was 80% with a 1-second epoch, 65% with a 15-second epoch (the validated epoch), and 55% with a 60-second epoch. Using the Evenson cut-point and Choi WT algorithm, minutes/day spent in MVPA was 17% greater with a 1-second epoch and 21% less with a 60-second epoch when compared to a 15-second epoch (the validated epoch). As shown, these differences in estimates may be large, potentially resulting in substantially different interpretations. Clinical and surveillance studies using different epoch lengths will therefore produce different estimates of individual and/or sample activity levels, potentially misleading researchers and policy makers who compare different demographic, geographic, and/or secular samples.

Our results are consistent with the limited existing literature for time spent in SB and PA intensity level from other samples. When examining the effects of converting a 100 count/60-second SB cutpoint and the Freedson activity cut-points among boys (14.4 ± 0.5 years) wearing an ActiGraph GT3X monitor for 4–7 days, Sanders and colleagues [6] found that boys spent 676.0 minutes/day in SB with a 1-second epoch, 556.9 minutes/day in SB with a 30-second epoch, and 525.0 minutes/day in SB with a 60-second epoch (the validated epoch). Similar results were observed for estimates of time spent in MVPA. When examining the effects of converting the Puyau activity cut-points among boys and girls (7 ± 2 years) wearing an ActiTrainer monitor for up to 7 days, Ojiambo and colleagues [8] found that children spent 24 minutes/day in MVPA with a 15-second epoch, 18 minutes/day in MVPA with a 30-second epoch, and 13 minutes/day in MVPA with a 60-second epoch (the validated epoch).

When comparing estimates of MVPA from direct observations of children (10.3 ± 0.5 years) in a 30-minute physical education class to estimates of MVPA from an ActiGraph 7164 monitor using different epoch lengths and activity cut-points, McClain and colleagues [18] found the smallest epoch length they examined (5-seconds) produced the smallest differences between direct observation-determined MVPA and Treuth cut-point and Mattocks cut-point determined MVPA. McClain and colleagues [18] recommended using shorter epoch lengths when possible due to the brief, intermittent nature of child PA [19, 20], and the smoothing of activity counts that can occur when an epoch length is longer than the bout of activity being measured [9]. However, they acknowledge that one must carefully weigh the potential benefit of using a smaller epoch length (e.g., 5-seconds) against the validity of modifying activity cut-point definitions (e.g., an activity cut-point validated at a 30-second epoch length) when deciding which epoch length to use. Because of the large differences in estimates of WT, SB, and levels of PA observed when converting the validated cut-point epochs, however, we recommend the use of the validation epoch length when possible and, at the very least, the reporting of data processing decisions to help the reader interpret the results. Their findings and ours highlight the need for additional WT algorithm and activity cut-point validation studies using smaller epoch lengths in children.

### Estimates of SB and PA by Activity Cut-point Definition

The large differences observed in estimates of SB and PA intensity levels between different activity cut-points are interesting as the activity cut-points were validated using similar methods (oxygen consumption was measured with a Cosmed model K4b2 metabolic unit in the Evenson [12], Treuth [13], Mattocks [15], and Romanzini [16] activity cut-points, and with a room calorimeter in the Puyau [14] activity cut-points) and designed to measure the same activity levels (i.e., SB, LPA, MPA, and VPA) in children.

Using the validated epoch length for each activity cut-point to compare their resulting estimates, minutes/day spent in SB when using the Choi WT algorithm ranged from 615.5 minutes/day to 849.5 minutes/day, while minutes/day spent in LPA ranged from 163.7 minutes/day to 388.4 minutes/day. Minutes/day spent in MVPA ranged from 15.2 minutes/day to 59.9 minutes/day for the four activity cut-points developed with vertical-axis counts. The upper limit of the MVPA range increases to 107.6 minutes/day when including the Romanzini activity cut-point, which was validated with vector magnitude counts. Similar large differences were observed between activity cut-points when examining minutes/day and percent time spent in SB, LPA, MPA, VPA, and MVPA at a particular epoch length (e.g., 15-second epoch) (all p values < .0001).

### Pairing Epoch Lengths, WT Algorithms, and Activity Cut-points

The results have important implications for the selection of activity cut-points that can be paired with different WT algorithms and epoch lengths. The ≥ 20 minute consecutive zero vertical-axis count and Choi WT algorithms may be paired with any epoch length without introducing significant differences in estimates of WT. As the NHANES WT algorithm yields different estimates of WT by epoch length (and it was validated with a 60-second epoch), it will produce different results in SB and PA levels when paired with an activity cut-point that does not use a 60-second epoch. For example, using the NHANES WT algorithm with data collected and/or analyzed at 15-second epoch results in a 4% increase in the estimate of average WT minutes/day in our sample. Although it is clear that different epoch lengths and algorithms and their combinations all provide different estimates, without a gold standard comparison there is no way to know which produces the most valid estimates.

Finally, it’s important to acknowledge that many studies do match the epoch length used in their data processing and analysis to the epoch length used in the validation of their chosen WT algorithm and activity cut-point definitions [21–23]. As just two examples of how researchers have done this, Engelen and colleagues [21] converted their data collected in 5-second epochs into 15-second epoch length data for analysis to match the epoch length used in the validation of their chosen activity cut-points, while Gortmaker and colleagues [22] applied the NHANES WT algorithm [3] and Freedson activity cut-points [24] (both developed for 60-second epoch length data) to match their 60-second epoch length data. By using the same epoch length used to validate the chosen WT algorithm and activity cut-point definitions, these studies have the benefit of not introducing this additional source of variation into their estimates, and allow comparisons with results from other studies using the same epoch lengths, WT algorithms, and activity cut-points.

### Strengths and Limitations

Strengths of this study include a large sample (N = 268 participants) of children who wore an accelerometer for at least 4–7 days. In addition, this study examined the effect of epoch length on WT, SB, and PA intensity levels using multiple commonly used WT algorithms and activity cut-points. While this study does not include a criterion measure, the activity cut-points used in this study were all validated using similar methods. As a result, the different activity cut-points would be expected to provide similar estimates of SB and PA intensity levels. However, this study found there are large differences in these estimates by activity cut-points, even above and beyond the effects of varying epoch lengths. Although the sample used was limited to primarily Hispanic or Latino children with overweight and obesity, our findings highlight features of accelerometer WT algorithms and activity level cut-points that apply to any sample being monitored with accelerometers. Finally, the results are limited to the WT algorithm and activity cut-point method of processing and analyzing accelerometer data, and to the WT algorithms and activity cut-point definitions examined in this study. However, the WT algorithms and activity cut-points examined in this study are commonly used in the literature, and WT algorithms and activity cut-points are a particularly common method of processing accelerometer data and determining time spent in SB and PA from accelerometers in children [1].

## Conclusion

This study found that three key decisions in accelerometer data processing and analysis might lead to substantially different results. We recommend first selecting a WT algorithm and activity cut-points that have been validated in a sample most closely matching the sample and methods used in the study of interest. We then recommend data processing and analysis using the same epoch length used to validate the chosen WT algorithm and activity cut-points, where possible. This approach will insure that resulting estimates will be comparable to other studies using the same epoch, WT algorithm, and activity cut-points. However, even when using the epoch length used in the original validation study, choice of activity cut-point can also produce widely varying results. At the very least, the specific epoch length, WT algorithm, and activity cut-points used should always be reported in studies to help readers interpret the results, and to avoid making direct comparisons between studies that use different methods.

We found that estimates of WT, SB, and PA intensity levels significantly vary by epoch length, and that using epoch lengths that differ from those originally used to validate the WT algorithms and activity cut-points introduces significant errors into resulting estimates of WT, SB, and PA intensity levels. These findings indicate that researchers, health and public health professionals, and policy makers cannot validly compare the amount of WT or time spent in SB, LPA, MPA, and VPA estimated from studies that use different epoch lengths, WT algorithms, and/or activity cut-points. The resulting differences in WT, SB, and levels of PA may be large, potentially resulting in substantially different results, interpretations, and conclusions. Clinical and public health research and surveillance studies using different epoch lengths, WT algorithms and/or activity cut-points will produce different estimates of individual and/or sample activity levels, potentially misleading policy makers who compare different demographic, geographic, and/or secular samples. The results of epidemiological studies examining associations between SB and PA with other behaviors and characteristics may also be highly sensitive to the epoch length, WT algorithm, and/or activity cut-point used. These differences can potentially produce very different results about SB and PA as correlates and/or risk factors for health, disease, and social outcomes. In experimental trials in which SB and PA are outcomes or mediating factors, the choice of epoch length, WT algorithm, and/or activity cut-point could affect estimated effect sizes, clouding the evidence base about intervention efficacy, and ultimately affecting the ability to create meaningful public health recommendations for SB and PA.

## Supporting Information

S1 Table WT, SB, and PA intensity levels by activity cut-point and epoch length using the ≥ 20 minute consecutive zero vertical-axis count WT algorithm. (DOCX)

S2 Table WT, SB, and PA intensity levels by activity cut-point and epoch length using the NHANES WT algorithm. (DOCX)

S3 Table WT, SB, and PA intensity levels by activity cut-point and epoch length using the Choi WT algorithm. (DOCX)

S4 Table SB and PA intensity levels by activity cut-point using the ≥ 20 minute consecutive zero vertical-axis count WT algorithm. (DOCX)

S5 Table SB and PA intensity levels by activity cut-point using the NHANES WT algorithm. (DOCX)

S6 Table SB and PA intensity levels activity cut-point using the Choi WT algorithm. (DOCX)
