# Supplementary material for: Effects of Varying Epoch Lengths, Wear Time Algorithms, and Activity Cut-Points on Estimates of Child Sedentary Behavior and Physical Activity from Accelerometer Data
Source: PLoS One. 2016 Mar 3;11(3):e0150534. doi: 10.1371/journal.pone.0150534 (PMC4777377; doi:10.1371/journal.pone.0150534)
Supplement: S1 Table — (DOCX) [file pone.0150534.s001.docx]

**S1 Table. WT, SB, and PA intensity levels by activity cut-point and epoch length using the ≥ 20 minute consecutive zero vertical-axis count WT algorithm.**

| Activity Cut-point | Epoch Length | WT | SB | LPA | MPA | VPA | MVPA |
| --- | --- | --- | --- | --- | --- | --- | --- |
|  | Second | Minute/day | Minute/Day  (% Time) | Minute/Day  (% Time) | Minute/Day  (% Time) | Minute/Day  (% Time) | Minute/Day  (% Time) |
| Evenson (12) | ANOVA | F(5,1335)=0.62  P=.6837 | F(5,1335)=2009.52  p<.0001  F(5,1335)=5527.62  p<.0001 | F(5,1335)=5608.83  p<.0001  F(5,1335)=10536.30  p<.0001 | F(5,1335)=48.60  p<.0001  F(5,1335)=55.50  p<.0001 | F(5,1335)=770.16  p<.0001  F(5,1335)=833.97  p<.0001 | F(5,1335)=233.30  p<.0001  F(5,1335)=266.18  p<.0001 |
|  | 1 | 927.77 | 720.06 ^^^^  (77.63%) ^^^^ | 136.41 ^^^^  (14.70%) ^^^^ | 39.33 ^^^^  (4.23%) ^^^^ | 31.91 ^^^^  (3.43%) ^^^^ | 71.25 ^^^^  (7.67%) ^^^^ |
|  | 5 | 928.16 | 631.97 ^^^^  (68.13%) ^^^^ | 228.02 ^^^^  (24.53%) ^^^^ | 43.08  (4.64%) | 25.03 ^^^^  (2.70%) ^^^^ | 68.12 ^^^^  (7.33%) ^^^^ |
|  | 10 | 928.66 | 583.44 ^^^^  (62.89%) ^^^^ | 280.91 ^^^^  (30.20%) ^^^^ | 43.83  (4.72%) | 20.42 ^^^^  (2.20%) ^^^^ | 64.25 ^^^^  (6.92%) ^^^^ |
|  | 15* | 929.17 | 556.39  (59.95%) | 311.77  (33.49%) | 43.23  (4.65%) | 17.73  (1.91%) | 60.96  (6.56%) |
|  | 30 | 930.78 | 505.59 ^^^^  (54.40%) ^^^^ | 370.79 ^^^^  (39.76%) ^^^^ | 40.65 ^^^^  (4.37%) ^^^^ | 13.70 ^^^^  (1.47%) ^^^^ | 54.35 ^^^^  (5.84%) ^^^^ |
|  | 60 | 933.66 | 455.89 ^^^^  (48.92%) ^^^^ | 429.86 ^^^^  (45.95%) ^^^^ | 37.53 ^^^^  (4.02%) ^^^^ | 10.33 ^^^^  (1.11%) ^^^^ | 47.86 ^^^^  (5.13%) ^^^^ |
| Treuth (13) | ANOVA | F(5,1335)=0.62  P=.6837 | F(5,1335)=2009.52  p<.0001  F(5,1335)=5527.62  p<.0001 | F(5,1335)=4930.93  p<.0001  F(5,1335)=9112.40  p<.0001 | F(5,1335)=179.08  p<.0001  F(5,1335)=199.00  p<.0001 | F(5,1335)=996.02  p<.0001  F(5,1335)=1056.65  p<.0001 | F(5,1335)=480.73  p<.0001  F(5,1335)=532.59  p<.0001 |
|  | 1 | 927.77 | 720.06 ^^^^  (77.63%) ^^^^ | 155.88 ^^^^  (16.80%) ^^^^ | 32.59 ^^^^  (3.50%) ^^^^ | 19.19 ^^^^  (2.07%) ^^^^ | 51.78 ^^^^  (5.57%) ^^^^ |
|  | 5 | 928.16 | 631.97 ^^^^  (68.13%) ^^^^ | 250.70 ^^^^  (26.98%) ^^^^ | 32.22 ^^^^  (3.46%) ^^^^ | 13.21 ^^^^  (1.42%) ^^^^ | 45.43 ^^^^  (4.89%) ^^^^ |
|  | 10 | 928.66 | 583.44 ^^^^  (62.89%) ^^^^ | 304.79 ^^^^  (32.77%) ^^^^ | 30.76 ^^^^  (3.31%) ^^^^ | 9.61 ^^^^  (1.04%) ^^^^ | 40.37 ^^^^  (4.34%) ^^^^ |
|  | 15 | 929.17 | 556.39 ^^^^  (59.95%) ^^^^ | 335.82 ^^^^  (36.08%) ^^^^ | 29.05 ^^^^  (3.12%) ^^^^ | 7.86 ^^^^  (0.85%) ^^^^ | 36.91 ^^^^  (3.97%) ^^^^ |
|  | 30* | 930.78 | 505.59  (54.40%) | 393.95  (42.25%) | 25.74  (2.76%) | 5.45  (0.59%) | 31.19  (3.35%) |
|  | 60 | 933.66 | 455.89 ^^^^  (48.92%) ^^^^ | 451.84 ^^^^  (48.31%) ^^^^ | 22.23 ^^^^  (2.38%) ^^^^ | 3.64 ^^^^  (0.39%) ^^^^ | 25.88 ^^^^  (2.77%) ^^^^ |
| Puyau (14) | ANOVA | F(5,1335)=0.62  P=.6837 | F(5,1335)=37.10  p<.0001  F(5,1335)=291.29  p<.0001 | F(5,1335)=925.20  p<.0001  F(5,1335)=1184.47  p<.0001 | F(5,1335)=386.99  p<.0001  F(5,1335)=425.46  p<.0001 | F(5,1335)=788.79  p<.0001  F(5,1335)=825.00  p<.0001 | F(5,1335)=516.05  p<.0001  F(5,1335)=568.22  p<.0001 |
|  | 1 | 927.77 | 784.81 ^^^^  (84.61%) ^^^^ | 96.83 ^^^^  (10.43%) ^^^^ | 39.28 ^^^^  (4.22%) ^^^^ | 6.82 ^^^^  (0.74%) ^^^^ | 46.10 ^^^^  (4.96%) ^^^^ |
|  | 5 | 928.16 | 766.05 ^^^^  (82.55%) ^^^^ | 121.82 ^^^^  (13.11%) ^^^^ | 37.12 ^^^^  (3.99%) ^^^^ | 3.15 ^^^^  (0.34%) ^^^^ | 40.27 ^^^^  (4.33%) ^^^^ |
|  | 10 | 928.66 | 757.41 ^^^  (81.57%) ^^^^ | 136.04 ^^^^  (14.64%) ^^^^ | 33.26 ^^^^  (3.58%) ^^^^ | 1.93 ^^^^  (0.21%) ^^^^ | 35.18 ^^^^  (3.79%) ^^^^ |
|  | 15 | 929.17 | 751.98  (80.95%) ^^^^ | 145.24 ^^^^  (15.62%) ^^^^ | 30.45 ^^^^  (3.27%) ^^^^ | 1.47 ^^^^  (0.16%) ^^^^ | 31.92 ^^^^  (3.43%) ^^^^ |
|  | 30 | 930.78 | 746.55  (80.22%) ^ | 157.73 ^^^^  (16.93%) ^^^^ | 25.48 ^^^^  (2.74%) ^^^^ | 0.99 ^  (0.11%) ^ | 26.47 ^^^^  (2.84%) ^^^^ |
|  | 60* | 933.66 | 745.90  (79.90%) | 166.07  (17.78%) | 20.95  (2.24%) | 0.71  (0.08%) | 21.66  (2.32%) |
| Mattocks (15) ** | ANOVA | F(5,1335)=0.62  P=.6837 | F(5,1335)=14.98  p<.0001  F(5,1335)=708.62  p<.0001 | | F(5,1335)=337.03  p<.0001  F(5,1335)=371.43  p<.0001 | F(5,1335)=1017.40  p<.0001  F(5,1335)=1068.75  p<.0001 | F(5,1335)=647.06  p<.0001  F(5,1335)=708.62  p<.0001 |
|  | 1 | 927.77 | 888.96 ^^^^  (95.83%) ^^^^ | | 25.34 ^^^^  (2.72%) ^^^^ | 13.46 ^^^^  (1.45%) ^^^^ | 38.80 ^^^^  (4.17%) ^^^^ |
|  | 5 | 928.16 | 896.03 ^^^^  (96.54%) ^^^^ | | 23.74 ^^^^  (2.55%) ^^^^ | 8.38 ^^^^  (0.91%) ^^^^ | 32.13 ^^^^  (3.46%) ^^^^ |
|  | 10 | 928.66 | 901.40 ^^^^  (97.07%) ^^^^ | | 21.63 ^^^^  (2.32%) ^^^^ | 5.64 ^^^^  (0.61%) ^^^^ | 27.26 ^^^^  (2.93%) ^^^^ |
|  | 15 | 929.17 | 905.04 ^^^  (97.40%) ^^^^ | | 19.70 ^^^^  (2.12%) ^^^^ | 4.43 ^^^^  (0.48%) ^^^^ | 24.13 ^^^^  (2.60%) ^^^^ |
|  | 30 | 930.78 | 911.36  (97.92%) ^^^^ | | 16.54 ^^^^  (1.77%) ^^^^ | 2.87 ^^^^  (0.31%) ^^^^ | 19.41 ^^^^  (2.08%) ^^^^ |
|  | 60* | 933.66 | 918.33  (98.36%) | | 13.47  (1.44%) | 1.86  (0.20%) | 15.33  (1.64%) |
| Romanzini (16) | ANOVA | F(5,1335)=0.62  P=.6837 | F(5,1335)=316.46  p<.0001  F(5,1335)=1013.71  p<.0001 | F(5,1335)=3052.69  p<.0001  F(5,1335)=5095.81  p<.0001 | F(5,1335)=62.07  p<.0001  F(5,1335)=77.72  p<.0001 | F(5,1335)=910.70  p<.0001  F(5,1335)=1022.94  p<.0001 | F(5,1335)=405.68  p<.0001  F(5,1335)=489.46  p<.0001 |
|  | 1 | 927.77 | 674.87 ^^^^  (72.79%) ^^^^ | 120.87 ^^^^  (13.00%) ^^^^ | 52.68 ^^^^  (5.67%) ^^^^ | 79.30 ^^^^  (8.54%) ^^^^ | 131.98 ^^^^  (14.21%) ^^^^ |
|  | 5 | 928.16 | 639.08 ^^^^  (68.92%) ^^^^ | 167.39 ^^^^  (17.98%) ^^^^ | 56.61 ^^^^  (6.09%) ^^^^ | 65.04 ^^^^  (7.01%) ^^^^ | 121.64 ^^^^  (13.10%) ^^^^ |
|  | 10 | 928.66 | 618.41 ^^^^  (66.66%) ^^^^ | 195.53 ^^^^  (20.99%) ^^^^ | 58.65  (6.31%) | 56.02 ^^^^  (6.04%) ^^^^ | 114.67 ^^^^  (12.35%) ^^^^ |
|  | 15* | 929.17 | 606.15  (65.31%) | 213.86  (22.94%) | 58.67  (6.31%) | 50.44  (5.44%) | 109.11  (11.75%) |
|  | 30 | 930.78 | 586.17 ^^^^  (63.05%) ^^^^ | 246.68 ^^^^  (26.42%) ^^^^ | 56.02 ^^^^  (6.02%) ^^^^ | 41.86 ^^^^  (4.51%) ^^^^ | 97.88 ^^^^  (10.53%) ^^^^ |
|  | 60 | 933.66 | 568.06 ^^^^  (60.91%) ^^^^ | 278.77 ^^^^  (29.77%) ^^^^ | 51.92 ^^^^  (5.57%) ^^^^ | 34.86 ^^^^  (3.75%) ^^^^ | 86.78 ^^^^  (9.31%) ^^^^ |

WT = Wear time, SB = Sedentary behavior, LPA = Light physical activity, MPA = Moderate physical activity, VPA = Vigorous physical activity

Minutes/day in SB, LPA, MPA, and VPA may not equal WT due to rounding. % Time spent in SB, LPA, MPA, and VPA may not equal 100% due to rounding. % Time spent in MPA and VPA may not equal MVPA due to rounding.

^ p < .05, ^^ p <.01, ^^^ p <.001, ^^^^ p <.0001 (significant pairwise difference in estimates of activity between the epoch length used to validate the activity cut-point and other epoch lengths).

* The epoch length used to derive the activity cut-points in the original validation studies.

** The Mattocks activity cut-point [14] does not provide separate activity cut-points for SB and LPA.
