# Supplementary material for: Effects of Varying Epoch Lengths, Wear Time Algorithms, and Activity Cut-Points on Estimates of Child Sedentary Behavior and Physical Activity from Accelerometer Data
Source: PLoS One. 2016 Mar 3;11(3):e0150534. doi: 10.1371/journal.pone.0150534 (PMC4777377; doi:10.1371/journal.pone.0150534)
Supplement: S2 Table — (DOCX) [file pone.0150534.s002.docx]

**S2 Table. WT, SB, and PA intensity levels by activity cut-point and epoch length using the NHANES WT algorithm.**

| Activity Cut-point | Epoch Length | WT | SB | LPA | MPA | VPA | MVPA |
| --- | --- | --- | --- | --- | --- | --- | --- |
|  | Second | Minutes/Day | Minutes/Day  (% Time) | Minutes/Day  (% Time) | Minutes/Day  (% Time) | Minutes/Day  (% Time) | Minutes/Day  (% Time) |
| Evenson (12) | ANOVA | F(5,1335)=56.41  p<.0001 | F(5,1335)=2394.18  p<.0001  F(5,1335)=5233.46  p<.0001 | F(5,1335)=4902.00  p<.0001  F(5,1335)=9276.75  p<.0001 | F(5,1335)=39.79  p<.0001  F(5,1335)=28.53  p<.0001 | F(5,1335)=686.55  p<.0001  F(5,1335)=648.15  p<.0001 | F(5,1335)=205.18  p<.0001  F(5,1335)=158.40  p<.0001 |
|  | 1 | 1030.33 | 824.56 ^^^^  (80.02%) ^^^^ | 135.94 ^^^^  (13.21%) ^^^^ | 39.03 ^^^^  (3.79%) ^^^^ | 30.74 ^^^^  (2.99%) ^^^^ | 69.77 ^^^^  (6.78%) ^^^^ |
|  | 5 | 1023.60 | 730.62 ^^^^  (71.39%) ^^^^ | 226.35 ^^^^  (22.09%) ^^^^ | 42.46  (4.15%) | 24.12 ^^^^  (2.36%) ^^^^ | 66.57 ^^^^  (6.51%) ^^^^ |
|  | 10 | 1016.82 | 675.50 ^^^^  (66.47%) ^^^^ | 278.47 ^^^^  (27.34%) ^^^^ | 43.10  (4.24%) | 19.70 ^^^^  (1.95%) ^^^^ | 62.80 ^^^^  (6.18%) ^^^ |
|  | 15 * | 1009.17 | 640.74  (63.54%) | 308.92  (30.55%) | 42.39  (4.20%) | 17.06  (1.70%) | 59.46  (5.90%) |
|  | 30 | 991.96 | 570.25 ^^^^  (57.56%) ^^^^ | 367.81 ^^^^  (37.00%) ^^^^ | 40.30 ^^^^  (4.07%) ^^ | 13.54 ^^^^  (1.37%) ^^^^ | 53.84 ^^^^  (5.44%) ^^^^ |
|  | 60 | 966.44 | 492.59 ^^^^  (51.07%) ^^^^ | 426.40 ^^^^  (44.02%) ^^^^ | 37.21 ^^^^  (3.85%) ^^^^ | 10.19 ^^^^  (1.06%) ^^^^ | 47.40 ^^^^  (4.91%) ^^^^ |
| Treuth (13) | ANOVA | F(5,1335)=56.41  p<.0001 | F(5,1335)=2394.18  p<.0001  F(5,1335)=5233.46  p<.0001 | F(5,1335)=4281.59  p<.0001  F(5,1335)=8038.57  p<.0001 | F(5,1335)=138.78  p<.0001  F(5,1335)=104.02  p<.0001 | F(5,1335)=914.34  p<.0001  F(5,1335)=874.86  p<.0001 | F(5,1335)=405.76 p <.0001  F(5,1335)=361.90  p<.0001 |
|  | 1 | 1030.33 | 824.56 ^^^^  (80.02%) ^^^^ | 155.86 ^^^^  (15.14%) ^^^^ | 31.35 ^^^^  (3.04%) ^^^^ | 18.50 ^^^^  (1.80%) ^^^^ | 49.85 ^^^^  (4.84%) ^^^^ |
|  | 5 | 1023.60 | 730.62 ^^^^  (71.39%) ^^^^ | 249.17 ^^^^  (24.32%) ^^^^ | 31.02 ^^^^  (3.03%) ^^^^ | 12.74 ^^^^  (1.25%) ^^^^ | 43.76 ^^^^  (4.28%) ^^^^ |
|  | 10 | 1016.82 | 675.50 ^^^^  (66.47%) ^^^^ | 302.31 ^^^^  (29.69%) ^^^^ | 29.67 ^^^^  (2.92%) ^^^^ | 9.29 ^^^^  (0.92%) ^^^^ | 38.96 ^^^^  (3.84%) ^^^^ |
|  | 15 | 1009.17 | 640.74 ^^^^  (63.54%) ^^^^ | 332.84 ^^^^  (32.92%) ^^^^ | 27.97 ^^^^  (2.77%) ^^^^ | 7.57 ^^^^  (0.76%) ^^^^ | 35.54 ^^^^  (3.53%) ^^^^ |
|  | 30* | 991.96 | 570.25  (57.56%) | 390.81  (39.32%) | 25.45  (2.57%) | 5.39  (0.55%) | 30.84  (3.12%) |
|  | 60 | 966.44 | 492.59 ^^^^  (51.07%) ^^^^ | 448.23 ^^^^  (46.28%) ^^^^ | 21.98 ^^^^  (2.27%) ^^^^ | 3.59 ^^^^  (0.38%) ^^^^ | 25.57 ^^^^  (2.65%) ^^^^ |
| Puyau (14) | ANOVA | F(5,1335)=56.41  p<.0001 | F(5,1335)=197.43  p<.0001  F(5,1335)=394.59  p<.0001 | F(5,1335)=819.11  p<.0001  F(5,1335)=1256.38  p<.0001 | F(5,1335)=323.71  p<.0001  F(5,1335)=281.95  p<.0001 | F(5,1335)=749.37  p<.0001  F(5,1335)=716.86  p<.0001 | F(5,1335)=441.29 p <.0001  F(5,1335)=398.75  p<.0001 |
|  | 1 | 1030.33 | 888.72^^^^  (86.25%) ^^^^ | 97.19 ^^^^  (9.43%) ^^^^ | 37.81 ^^^^  (3.67%) ^^^^ | 6.58 ^^^^  (0.64%) ^^^^ | 44.39 ^^^^  (4.31%) ^^^^ |
|  | 5 | 1023.60 | 863.09 ^^^^  (84.32%) ^^^^ | 121.70 ^^^^  (11.88%) ^^^^ | 35.75 ^^^^  (3.49%) ^^^^ | 3.04 ^^^^ (0.30%) ^^^^ | 38.79 ^^^^  (3.80%) ^^^^ |
|  | 10 | 1016.82 | 847.26 ^^^^  (83.33%) ^^^^ | 135.58 ^^^^  (13.32%) ^^^^ | 32.09 ^^^^  (3.16%) ^^^^ | 1.86 ^^^^  (0.19%) ^^^^ | 33.95 ^^^^  (3.35%) ^^^^ |
|  | 15 | 1009.17 | 833.82 ^^^^  (82.63%) ^^^^ | 144.58 ^^^^  (14.31%) ^^^^ | 29.33 ^^^^  (2.91%) ^^^^ | 1.42 ^^^^  (0.14%) ^^^^ | 30.74 ^^^^ (3.06%) ^^^^ |
|  | 30 | 991.96 | 809.22 ^^^^  (81.59%) ^^^^ | 156.54 ^^^^  (15.76%) ^^^^ | 25.19 ^^^^  (2.54%) ^^^^ | 0.98 ^  (0.10%) ^ | 26.17 ^^^^  (2.64%) ^^^^ |
|  | 60* | 966.44 | 780.22  (80.75%) | 164.79  (17.03%) | 20.70  (2.14%) | 0.70  (0.07%) | 21.40  (2.22%) |
| Mattocks (15) ** | ANOVA | F(5,1335)=56.41  p<.0001 | F(5,1335)=28.46  p<.0001  F(5,1335)=527.07  p<.0001 | | F(5,1335)=283.58  p<.0001  F(5,1335)=246.38  p<.0001 | F(5,1335)=947.29  p<.0001  F(5,1335)=906.34  p<.0001 | F(5,1335)=566.35 p <.0001  F(5,1335)=527.08  p<.0001 |
|  | 1 | 1030.33 | 992.96 ^^^^  (96.37%) ^^^^ | | 24.38 ^^^^  (2.36%) ^^^^ | 12.98 ^^^^  (1.26%) ^^^^ | 37.36 ^^^^  (3.63%) ^^^^ |
|  | 5 | 1023.60 | 992.65 ^^^^  (96.97%) ^^^^ | | 22.87 ^^^^  (2.23%) ^^^^ | 8.08 ^^^^  (0.80%) ^^^^ | 30.95 ^^^^  (3.03%) ^^^^ |
|  | 10 | 1016.82 | 990.51 ^^^^  (97.41%) ^^^^ | | 20.86 ^^^^  (2.05%) ^^^^ | 5.44 ^^^^  (0.54%) ^^^^ | 26.30 ^^^^  (2.59%) ^^^^ |
|  | 15 | 1009.17 | 985.94 ^^^^  (97.69%) ^^^^ | | 18.96 ^^^^  (1.88%) ^^^^ | 4.26 ^^^^  (0.43%) ^^^^ | 23.23 ^^^^  (2.31%) ^^^^ |
|  | 30 | 991.96 | 972.77 ^^^^  (98.06%) ^^^^ | | 16.35 ^^^^  (1.65%) ^^^^ | 2.84 ^^^^  (0.29%) ^^^^ | 19.19 ^^^^  (1.94%) ^^^^ |
|  | 60* | 966.44 | 951.30  (98.43%) | | 13.31  (1.38%) | 1.83  (0.19%) | 15.14  (1.57%) |
| Romanzini (16) | ANOVA | F(5,1335)=56.41  p<.0001 | F(5,1335)=644.65  p<.0001  F(5,1335)=1168.16  p<.0001 | F(5,1335)=2684.53  p<.0001  F(5,1335)=4782.63  p<.0001 | F(5,1335)=57.82  p<.0001  F(5,1335)=56.69  p<.0001 | F(5,1335)=877.61  p<.0001  F(5,1335)=823.61  p<.0001 | F(5,1335)=382.17  p<.0001  F(5,1335)=315.59  p<.0001 |
|  | 1 | 1030.33 | 779.41 ^^^^  (75.66%) ^^^^ | 119.95 ^^^^  (11.64%) ^^^^ | 52.25 ^^^^  (5.07%) ^^^^ | 78.66 ^^^^  (7.63%) ^^^^ | 130.91 ^^^^  (12.70%) ^^^^ |
|  | 5 | 1023.60 | 736.88 ^^^^  (72.02%) ^^^^ | 166.01 ^^^^  (16.19%) ^^^^ | 56.14 ^^^^  (5.48%) ^^^^ | 64.52 ^^^^  (6.31%) ^^^^ | 120.65 ^^^^  (11.79%) ^^^^ |
|  | 10 | 1016.82 | 709.14 ^^^^  (69.78%) ^^^^ | 193.88 ^^^^  (19.02%) ^^^^ | 58.16  (5.72%) | 55.57 ^^^^  (5.48%) ^^^^ | 113.74 ^^^^  (11.19%) ^^^^ |
|  | 15* | 1009.17 | 688.86  (68.31%) | 212.02  (20.95%) | 58.19  (5.77%) | 50.04  (4.97%) | 108.23  (10.74%) |
|  | 30 | 991.96 | 650.25 ^^^^  (65.60%) ^^^^ | 244.56 ^^^^  (24.59%) ^^^^ | 55.55 ^^^^  (5.61%) ^^ | 41.53 ^^^^  (4.20%) ^^^^ | 97.09 ^^^^  (9.81%) ^^^^ |
|  | 60 | 966.44 | 603.91 ^^^^  (62.55%) ^^^^ | 276.40 ^^^^  (28.53%) ^^^^ | 51.48 ^^^^  (5.33%) ^^^^ | 34.60 ^^^^  (3.59%) ^^^^ | 86.08 ^^^^  (8.92%) ^^^^ |

WT = Wear time, SB = Sedentary behavior, LPA = Light physical activity, MPA = Moderate physical activity, VPA = Vigorous physical activity

Minutes/day in SB, LPA, MPA, and VPA may not equal WT due to rounding. % Time spent in SB, LPA, MPA, and VPA may not equal 100% due to rounding. % Time spent in MPA and VPA may not equal MVPA due to rounding.

^ p < .05, ^^ p <.01, ^^^ p <.001, ^^^^ p <.0001 (significant pairwise difference in estimates of activity between the epoch length used to validate the activity cut-point and other epoch lengths).

* The epoch length used to derive the activity cut-points in the original validation studies.

** The Mattocks activity cut-point [14] does not provide separate activity cut-points for SB and LPA.
