# Supplementary material for: Effects of Varying Epoch Lengths, Wear Time Algorithms, and Activity Cut-Points on Estimates of Child Sedentary Behavior and Physical Activity from Accelerometer Data
Source: PLoS One. 2016 Mar 3;11(3):e0150534. doi: 10.1371/journal.pone.0150534 (PMC4777377; doi:10.1371/journal.pone.0150534)
Supplement: S3 Table — (DOCX) [file pone.0150534.s003.docx]

**S3 Table. WT, SB, and PA intensity levels by activity cut-point and epoch length using the Choi WT algorithm.**

| Activity Cut-point | Epoch Length | WT | SB | LPA | MPA | VPA | MVPA |
| --- | --- | --- | --- | --- | --- | --- | --- |
|  | Second | Minutes/Day | Minutes/Day  (% Time) | Minutes/Day  (% Time) | Minutes/Day  (% Time) | Minutes/Day  (% Time) | Minutes/Day  (% Time) |
| Evenson (12) | ANOVA | F(5,1335)=0  P=1.00 | F(5,1335)=1451.96  p<.0001  F(5,1335)=3933.50  p<.0001 | F(5,1335)=4492.39  p<.0001  F(5,1335)=7307.75  p<.0001 | F(5,1335)=44.21  p<.0001  F(5,1335)=45.93  p<.0001 | F(5,1335)=740.61  p<.0001  F(5,1335)=738.33  p<.0001 | F(5,1335)=217.79  p <.0001  F(5,1335)=227.44  p<.0001 |
|  | 1 | 1034.65 | 829.62 ^^^^  (80.21%) ^^^^ | 134.75 ^^^^  (13.01%) ^^^^ | 38.78 ^^^^  (3.74%) ^^^^ | 31.46 ^^^^  (3.04%) ^^^^ | 70.24 ^^^^  (6.78%) ^^^^ |
|  | 5 | 1034.65 | 742.51 ^^^^  (71.84%) ^^^^ | 224.94 ^^^^  (21.67%) ^^^^ | 42.47  (4.10%) | 24.69 ^^^^  (2.39%) ^^^^ | 67.17 ^^^^  (6.49%) ^^^^ |
|  | 10 | 1034.65 | 694.23 ^^^^  (67.20%) ^^^^ | 277.02 ^^^^  (26.67%) ^^^^ | 43.21  (4.17%) | 20.15 ^^^^  (1.96%) ^^^^ | 63.36 ^^^^  (6.13%) ^^^^ |
|  | 15* | 1034.65 | 667.51  (64.63%) | 307.25  (29.58%) | 42.45  (4.10%) | 17.40  (1.69%) | 59.85  (5.79%) |
|  | 30 | 1034.65 | 615.47 ^^^^  (59.63%) ^^^^ | 365.52 ^^^^  (35.17%) ^^^^ | 40.07 ^^^^  (3.88%) ^^^^ | 13.54 ^^^^  (1.32%) ^^^^ | 53.62 ^^^^  (5.20%) ^^^^ |
|  | 60 | 1034.65 | 563.69 ^^^^  (54.65%) ^^^^ | 423.70 ^^^^  (40.77%) ^^^^ | 37.01 ^^^^  (3.58%) ^^^^ | 10.21 ^^^^  (1.00%) ^^^^ | 47.23 ^^^^  (4.58%) ^^^^ |
| Treuth (13) | ANOVA | F(5,1335)=0  P=1.00 | F(5,1335)=1451.96  p<.0001  F(5,1335)=3933.50  p<.0001 | F(5,1335)=3953.14  p<.0001  F(5,1335)=6338.28  p<.0001 | F(5,1335)=168.18  p<.0001  F(5,1335)=171.46  p<.0001 | F(5,1335)=967.38  p<.0001  F(5,1335)=936.48  p<.0001 | F(5,1335)=455.29  p <.0001  F(5,1335)=466.01  p<.0001 |
|  | 1 | 1034.65 | 829.62 ^^^^  (80.21%) ^^^^ | 153.95 ^^^^  (14.86%)^^^^ | 32.12 ^^^^  (3.10%) ^^^^ | 18.92 ^^^^ (1.83%)^^^^ | 51.04 ^^^^  (4.93%) ^^^^ |
|  | 5 | 1034.65 | 742.51 ^^^^  (71.84%) ^^^^ | 247.30 ^^^^  (23.83%)^^^^ | 31.76 ^^^^  (3.06%) ^^^^ | 13.04 ^^^^  (1.27%)^^^^ | 44.80 ^^^^  (4.33%) ^^^^ |
|  | 10 | 1034.65 | 694.23 ^^^^  (67.20%) ^^^^ | 300.56 ^^^^  (28.94%) ^^^^ | 30.32 ^^^^  (2.93%) ^^^^ | 9.50 ^^^^  (0.93%)^^^^ | 39.82 ^^^^  (3.85%) ^^^^ |
|  | 15 | 1034.65 | 667.51 ^^^^  (64.63%) ^^^^ | 330.91 ^^^^  (31.86%) ^^^^ | 28.46 ^^^^  (2.75%) ^^^^ | 7.73 ^^^^  (0.76%) ^^^^ | 36.19 ^^^^  (3.51%) ^^^^ |
|  | 30* | 1034.65 | 615.47  (59.63%) | 388.36  (37.38%) | 25.38  (2.46%) | 5.39  (0.53%) | 30.78  (2.99%) |
|  | 60 | 1034.65 | 563.69 ^^^^  (54.65%) ^^^^ | 445.36 ^^^^  (42.87%) ^^^^ | 21.94 ^^^^  (2.13%) ^^^^ | 3.61 ^^^^  (0.36%) ^^^^ | 25.56 ^^^^  (2.49%) ^^^^ |
| Puyau (14) | ANOVA | F(5,1335)=0  P=1.00 | F(5,1335)=32.65  p<.0001  F(5,1335)=253.36  p<.0001 | F(5,1335)=797.54  p<.0001  F(5,1335)=996.28  p<.0001 | F(5,1335)=346.35  p<.0001  F(5,1335)=348.64  p<.0001 | F(5,1335)=780.48  p<.0001  F(5,1335)=805.03  p<.0001 | F(5,1335)=470.07  p <.0001  F(5,1335)=479.27  p<.0001 |
|  | 1 | 1034.65 | 893.72 ^^^^  (86.40%) ^^^^ | 95.81 ^^^^  (9.24%) ^^^^ | 38.41 ^^^^  (3.71%) ^^^^ | 6.68 ^^^^  (0.65%) ^^^^ | 45.09 ^^^^  (4.36%) ^^^^ |
|  | 5 | 1034.65 | 874.89 ^^^^  (84.59%) ^^^^ | 120.32 ^^^^  (11.60%) ^^^^ | 36.33 ^^^^  (3.51%) ^^^^ | 3.09 ^^^^  (0.30%) ^^^^ | 39.42 ^^^^  (3.81%) ^^^^ |
|  | 10 | 1034.65 | 866.10 ^^^^  (83.74%) ^^^^ | 134.75 ^^^^  (12.99%) ^^^^ | 31.93 ^^^^  (3.09%) ^^^^ | 1.85 ^^^^  (0.18%) ^^^^ | 33.78 ^^^^  (3.27%) ^^^^ |
|  | 15 | 1034.65 | 860.33 ^^  (83.18%) ^^^^ | 143.70 ^^^^  (13.86%) ^^^^ | 29.19 ^^^^  (2.83%) ^^^^ | 1.41 ^^^^  (0.14%) ^^^^ | 30.60 ^^^^  (2.96%) ^^^^ |
|  | 30 | 1034.65 | 852.99  (82.47%) ^ | 155.54 ^^^^  (14.99%) ^^^^ | 25.13 ^^^^  (2.44%) ^^^^ | 0.97 ^  (0.10%) ^ | 26.10 ^^^^  (2.53%) ^^^^ |
|  | 60* | 1034.65 | 849.53  (82.13) | 163.73  (15.79%) | 20.67  (2.01%) | 0.70  (0.07%) | 21.37  (2.08%) |
| Mattocks (15) ** | ANOVA | F(5,1335)=0  P=1.00 | F(5,1335)=6.86  p<.0001  F(5,1335)=625.21  p<.0001 | | F(5,1335)=320.86  p<.0001  F(5,1335)=326.16  p<.0001 | F(5,1335)=995.50  p<.0001  F(5,1335)=967.04  p<.0001 | F(5,1335)=618.82  p <.0001  F(5,1335)=625.21  p<.0001 |
|  | 1 | 1034.65 | 996.40 ^^^^  (96.31%) ^^^^ | | 24.98 ^^^^  (2.41%) ^^^^ | 13.27 ^^^^  (1.29%) ^^^^ | 38.25 ^^^^  (3.69%) ^^^^ |
|  | 5 | 1034.65 | 1002.97 ^^^  (96.93%) ^^^^ | | 23.41 ^^^^  (2.26%) ^^^^ | 8.28 ^^^^  (0.81%) ^^^^ | 31.68 ^^^^  (3.07%) ^^^^ |
|  | 10 | 1034.65 | 1007.75 ^^  (97.39%) ^^^^ | | 21.32 ^^^^  (2.06%) ^^^^ | 5.57 ^^^^  (0.55%) ^^^^ | 26.90 ^^^^  (2.61%) ^^^^ |
|  | 15 | 1034.65 | 1010.84  (97.69%) ^^^^ | | 19.43 ^^^^  (1.88%) ^^^^ | 4.38 ^^^^  (0.43%) ^^^^ | 23.81 ^^^^  (2.31%) ^^^^ |
|  | 30 | 1034.65 | 1015.48  (98.14%) ^^^^ | | 16.33 ^^^^  (1.58%) ^^^^ | 2.84 ^^^^  (0.28%) ^^^^ | 19.17 ^^^^  (1.86%) ^^^^ |
|  | 60* | 1034.65 | 1019.49  (98.52%) | | 13.31  (1.29%) | 1.84  (0.18%) | 15.15  (1.48%) |
| Romanzini (16) | ANOVA | F(5,1335)=0  P=1.00 | F(5,1335)=244.89  p<.0001  F(5,1335)=759.40  p<.0001 | F(5,1335)=2463.06  p<.0001  F(5,1335)=3687.01  p<.0001 | F(5,1335)=55.03  p<.0001  F(5,1335)=63.85  p<.0001 | F(5,1335)=849.52  p<.0001  F(5,1335)=896.48  p<.0001 | F(5,1335)=365.01  p<.0001  F(5,1335)=406.80  p<.0001 |
|  | 1 | 1034.65 | 785.22 ^^^^  (75.96%) ^^^^ | 119.31 ^^^^  (11.49%) ^^^^ | 51.93 ^^^^  (5.00%) ^^^^ | 78.15 ^^^^  (7.54%) ^^^^ | 130.08 ^^^^  (12.55%) ^^^^ |
|  | 5 | 1034.65 | 749.66 ^^^^  (72.55%) ^^^^ | 165.06 ^^^^  (15.88%) ^^^^ | 55.79 ^^^  (5.38%) ^^^^ | 64.11 ^^^^  (6.20%) ^^^^ | 119.90 ^^^^  (11.57%) ^^^^ |
|  | 10 | 1034.65 | 728.85 ^^^  (70.55%) ^^^^ | 192.72 ^^^^  (18.53%) ^^^^ | 57.81  (5.57%) | 55.23 ^^^^  (5.35%) ^^^^ | 113.04 ^^^^  (10.92%) ^^^^ |
|  | 15* | 1034.65 | 716.28  (69.34%) | 210.76  (20.26%) | 57.83  (5.58%) | 49.74  (4.82%) | 107.57  (10.40%) |
|  | 30 | 1034.65 | 695.03 ^^^^  (67.30%) ^^^^ | 243.06 ^^^^  (23.36%) ^^^^ | 55.22 ^^^^  (5.33%) ^^^^ | 41.29 ^^^^  (4.01%) ^^^^ | 96.51 ^^^^  (9.34%) ^^^^ |
|  | 60 | 1034.65 | 674.35 ^^^^  (65.30%) ^^^^ | 274.67 ^^^^  (26.40%) ^^^^ | 51.18 ^^^^  (4.94%) ^^^^ | 34.40 ^^^^  (3.35%) ^^^^ | 85.58 ^^^^  (8.29%) ^^^^ |

WT = Wear time, SB = Sedentary behavior, LPA = Light physical activity, MPA = Moderate physical activity, VPA = Vigorous physical activity

Minutes/day in SB, LPA, MPA, and VPA may not equal WT due to rounding. % Time spent in SB, LPA, MPA, and VPA may not equal 100% due to rounding. % Time spent in MPA and VPA may not equal MVPA due to rounding.

^ p < .05, ^^ p <.01, ^^^ p <.001, ^^^^ p <.0001 (significant pairwise difference in estimates of activity between the epoch length used to validate the activity cut-point and other epoch lengths).

* The epoch length used to derive the activity cut-points in the original validation studies.

** The Mattocks activity cut-point [14] does not provide separate activity cut-points for SB and LPA.
