# Supplementary material for: Effects of Varying Epoch Lengths, Wear Time Algorithms, and Activity Cut-Points on Estimates of Child Sedentary Behavior and Physical Activity from Accelerometer Data
Source: PLoS One. 2016 Mar 3;11(3):e0150534. doi: 10.1371/journal.pone.0150534 (PMC4777377; doi:10.1371/journal.pone.0150534)
Supplement: S4 Table — (DOCX) [file pone.0150534.s004.docx]

**S4 Table. SB and PA intensity levels by activity cut-point using the ≥ 20 minute consecutive zero vertical-axis count WT algorithm.**

| Activity Cut-point | Epoch Length used  in Validation Study | SB | LPA | MPA | VPA | MVPA |
| --- | --- | --- | --- | --- | --- | --- |
|  | Second | Minute/Day  (% Time) | Minute/Day  (% Time) | Minute/Day  (% Time) | Minute/Day  (% Time) | Minute/Day  (% Time) |
|  | ANOVA | F(3,801)= 2230.00  p<.0001  F(3,801)= 6000.89  p<.0001 | F(3,801)= 5343.87  p<.0001  F(3,801)= 9038.16  p<.0001 | F(4,1068)= 2604.51  p<.0001  F(4,1068)= 2873.39  p<.0001 | F(4,1068)= 3724.79  p<.0001  F(4,1068)= 3900.04  p<.0001 | F(4,1068)= 4406.37  p<.0001  F(4,1068)= 4861.34  p<.0001 |
| Evenson (12) | 15 | 556.36  (59.95%) | 311.88  (33.50%) | 43.19  (4.65%) | 17.69  (1.90%) | 60.88  (6.55%) |
| Treuth (13) | 30 | 505.55  (54.40%) | 393.97  (42.24%) | 25.73  (2.76%) | 5.48  (0.59%) | 31.21  (3.35%) |
| Puyau (14) | 60 | 745.71  (79.86%) | 166.20  (17.81%) | 20.92  (2.24%) | 0.79  (0.09%) | 21.71  (2.33%) |
| Mattocks (15) * | 60 | . | . | 13.50  (1.44%) | 1.91  (0.21%) | 15.41  (1.65%) |
| Romanzini (16) | 15 | 606.19  (65.31%) | 213.97  (22.96%) | 58.68  (6.31%) | 50.28  (5.41%) | 108.96  (11.72%) |

WT = Wear time, SB = Sedentary behavior, LPA = Light physical activity, MPA = Moderate physical activity, VPA = Vigorous physical activity

% Time spent in SB, LPA, MPA, and VPA may not equal 100% due to rounding. % Time spent in MPA and VPA may not equal MVPA due to rounding.

* The Mattocks activity cut-point [14] does not provide separate activity cut-points for SB and LPA.

All pairwise comparisons for minutes per day and percent time spent in SB, LPA, MPA, VPA, and MVPA between activity cut-points were significant at p < .0001 except for VPA minutes per day (p = .0196) and percent time (p = .0175) spent in VPA between the Mattocks and Puyau activity cut-points.
