# Supplementary material for: Effects of Varying Epoch Lengths, Wear Time Algorithms, and Activity Cut-Points on Estimates of Child Sedentary Behavior and Physical Activity from Accelerometer Data
Source: PLoS One. 2016 Mar 3;11(3):e0150534. doi: 10.1371/journal.pone.0150534 (PMC4777377; doi:10.1371/journal.pone.0150534)
Supplement: S5 Table — (DOCX) [file pone.0150534.s005.docx]

**S5 Table. SB and PA intensity levels by activity cut-point using the NHANES WT algorithm.**

| Activity Cut-point | Epoch Length used  in Validation Study | SB | LPA | MPA | VPA | MVPA |
| --- | --- | --- | --- | --- | --- | --- |
|  | Second | Minutes/Day  (% Time) | Minutes/Day  (% Time) | Minutes/Day  (% Time) | Minutes/Day  (% Time) | Minutes/Day  (% Time) |
|  | ANOVA | F(3,801)= 1254.41  p<.0001  F(3,801)= 4695.52  p<.0001 | F(3,801)= 4761.58  p<.0001  F(3,801)= 7280.33  p<.0001 | F(4,1068)= 2482.87  p<.0001  F(4,1068)= 2519.82  p<.0001 | F(4,1068)= 3653.03  p<.0001  F(4,1068)= 3727.91  p<.0001 | F(4,1068)= 4215.17  p<.0001  F(4,1068)= 4402.83  p<.0001 |
| Evenson (12) | 15 | 640.89  (63.54%) | 308.99  (30.56%) | 42.34  (4.20%) | 17.07  (1.70%) | 59.41  (5.90%) |
| Treuth (13) | 30 | 570.40  (57.56%) | 390.74  (39.31%) | 25.50  (2.57%) | 5.43  (0.55%) | 30.94  (3.13%) |
| Puyau (14) | 60 | 780.13  (80.70%) | 164.90  (17.06%) | 20.74  (2.15%) | 0.78  (0.09%) | 21.53  (2.23%) |
| Mattocks (15) * | 60 | . | . | 13.40  (1.39%) | 1.89  (0.20%) | 15.30  (1.59%) |
| Romanzini (16) | 15 | 689.03  (68.31%) | 212.28  (20.98%) | 58.15  (5.76%) | 49.82  (4.94%) | 107.98  (10.70%) |

WT = Wear time, SB = Sedentary behavior, LPA = Light physical activity, MPA = Moderate physical activity, VPA = Vigorous physical activity

% Time spent in SB, LPA, MPA, and VPA may not equal 100% due to rounding. % Time spent in MPA and VPA may not equal MVPA due to rounding.

* The Mattocks activity cut-point [14] does not provide separate activity cut-points for SB and LPA.

All pairwise comparisons for minutes per day and percent time spent in SB, LPA, MPA, VPA, and MVPA between activity cut-points were significant at p < .0001 except for VPA minutes per day (p = .0206) and percent time (p = .0144) spent in VPA between the Mattocks and Puyau activity cut-points.
