# Supplementary material for: Effects of Varying Epoch Lengths, Wear Time Algorithms, and Activity Cut-Points on Estimates of Child Sedentary Behavior and Physical Activity from Accelerometer Data
Source: PLoS One. 2016 Mar 3;11(3):e0150534. doi: 10.1371/journal.pone.0150534 (PMC4777377; doi:10.1371/journal.pone.0150534)
Supplement: S6 Table — (DOCX) [file pone.0150534.s006.docx]

**S6 Table. SB and PA intensity levels activity cut-point using the Choi WT algorithm.**

| Activity Cut-point | Epoch Length used in Validation Study | SB | LPA | MPA | VPA | MVPA |
| --- | --- | --- | --- | --- | --- | --- |
|  | Second | Minutes/Day  (% Time) | Minutes/Day  (% Time) | Minutes/Day  (% Time) | Minutes/Day  (% Time) | Minutes/Day  (% Time) |
|  | ANOVA | F(3,801)= 1521.63  p<.0001  F(3,801)= 4381.15  p<.0001 | F(3,801)= 4388.25  p<.0001  F(3,801)= 6622.28  p<.0001 | F(4,1068)= 2428.44  p<.0001  F(4,1068)= 2516.78  p<.0001 | F(4,1068)= 3616.58  p<.0001  F(4,1068)= 3631.84  p<.0001 | F(4,1068)= 4123.57  p<.0001  F(4,1068)= 4313.93  p<.0001 |
| Evenson (12) | 15 | 667.54  (64.63%) | 307.31  (29.58%) | 42.38  (4.09%) | 17.36  (1.69%) | 59.74  (5.78%) |
| Treuth (13) | 30 | 615.51  (59.63%) | 388.29  (37.37%) | 25.38  (2.46%) | 5.42  (0.53%) | 30.81  (2.99%) |
| Puyau (14) | 60 | 849.13  (82.07%) | 164.01  (15.83%) | 20.67  (2.01%) | 0.78  (0.08%) | 21.46  (2.09%) |
| Mattocks (15) * | 60 | . | . | 13.36  (1.30%) | 1.90  (0.19%) | 15.26  (1.49%) |
| Romanzini (16) | 15 | 716.21  (69.33%) | 211.06  (20.30%) | 57.78  (5.57%) | 49.54  (4.79%) | 107.33  (10.36%) |

WT = Wear time, SB = Sedentary behavior, LPA = Light physical activity, MPA = Moderate physical activity, VPA = Vigorous physical activity

% Time spent in SB, LPA, MPA, and VPA may not equal 100% due to rounding. % Time spent in MPA and VPA may not equal MVPA due to rounding.

* The Mattocks activity cut-point [14] does not provide separate activity cut-points for SB and LPA.

All pairwise comparisons for minutes per day and percent time spent in SB, LPA, MPA, VPA, and MVPA between activity cut-points were significant at p < .0001 except for VPA minutes per day (p = .0203) and percent time (p = .0182) spent in VPA between the Mattocks and Puyau activity cut-points.
